# Russian Science Directive Leaves Researchers Cold

**DOI:** 10.1100/tsw.2001.59

**Published:** 2001-06-08

**Authors:** Shauna M. Haley

## Abstract

Both Nature and Science lead the news week with coverage of Russia's new science directive: that all contacts made by Russian researchers with foreigners be reported in detail.

On the last day of May, the Russian government announced that all international collaboration contacts made by Russian scientists will now require full report by those scientists. The premise of the order is to prevent the theft of state secrets through espionage, says *Nature*. The directive has not hampered travel or the practice of research yet, note several scientists in both journals. The move could be a method for the Russian Academy of Sciences (RAS) to regain lost control over its staff, says biophysicist Maxim Frank-Kamenetskii in *Nature*. Many current projects participated in by Russian scientists are funded by international grants. *Science* reports that the new order also requires scientists to submit original research articles to the RAS for approval when submitting to foreign journals. Snarling over the rules ensued because no one is sure where the new directive originated, says *Science*. Some observers suspect that the Federal Security Service (who succeeded the KGB) is trying to exert control over scientists in a return to a Soviet security style, while others speculate that the real pinch on scientists could come through each science institute's interpretive enforcement of the rules. In either case, scientists and investors all wait to exhale while the carried-out directive begins to take shape.

Also among the top stories in *Nature* and *Science* this week are reports of the quitting of the U.S. Smithsonian Institution's Natural History Museum director over the continued controversial restructuring of the Institute, and of an international fight centered on the patenting of the now-famous breast cancer gene, the Philadelphia chromosome.
